# A path to precision in the ICU

**DOI:** 10.1186/s13054-017-1653-x

**Published:** 2017-04-03

**Authors:** David M. Maslove, Francois Lamontagne, John C. Marshall, Daren K. Heyland

**Affiliations:** 1 Department of Critical Care Medicine, Queen’s University, Kingston, ON Canada; 2 Department of Medicine, Queen’s University, Kingston, ON Canada; 3 Department of Critical Care Medicine, Kingston General Hospital, Davies 2, 76 Stuart St., Kingston, Ontario K7L 2V7 Canada; 4 Department of Medicine, Université de Sherbrooke, Sherbrooke, QC Canada; 5 Centre de Recherche du CHU de Sherbrooke, Sherbrooke, QC Canada; 6 Centre Hospitalier Universitaire de Sherbrooke, Sherbrooke, QC Canada; 7 Department of Surgery, University of Toronto, Toronto, ON Canada; 8 Interdepartmental Division of Critical Care, University of Toronto, Toronto, ON Canada; 9 St. Michael’s Hospital, Toronto, ON Canada; 10 Clinical Evaluation Research Unit, Kingston General Hospital, Kingston, ON Canada

**Keywords:** Precision medicine, Precision health, Personalized medicine, Clinical trials, Genomics, Biomedical informatics, Critical care, Intensive care unit

## Abstract

Precision medicine is increasingly touted as a groundbreaking new paradigm in biomedicine. In the ICU, the complexity and ambiguity of critical illness syndromes have been identified as fundamental justifications for the adoption of a precision approach to research and practice. Inherently protean diseases states such as sepsis and acute respiratory distress syndrome have manifestations that are physiologically and anatomically diffuse, and that fluctuate over short periods of time. This leads to considerable heterogeneity among patients, and conditions in which a “one size fits all” approach to therapy can lead to widely divergent results. Current ICU therapy can thus be seen as *imprecise*, with the potential to realize substantial gains from the adoption of precision medicine approaches. A number of challenges still face the development and adoption of precision critical care, a transition that may occur incrementally rather than wholesale. This article describes a few concrete approaches to addressing these challenges.

First, novel clinical trial designs, including registry randomized controlled trials and platform trials, suggest ways in which conventional trials can be adapted to better accommodate the physiologic heterogeneity of critical illness. Second, beyond the “omics” technologies already synonymous with precision medicine, the data-rich environment of the ICU can generate complex physiologic signatures that could fuel precision-minded research and practice. Third, the role of computing infrastructure and modern informatics methods will be central to the pursuit of precision medicine in the ICU, necessitating close collaboration with data scientists. As work toward precision critical care continues, small proof-of-concept studies may prove useful in highlighting the potential of this approach.

## Background

Spurred by advances in genomics and big data analytics, precision medicine is increasingly touted as a groundbreaking new paradigm in biomedicine. Broadly construed, the term “precision medicine” describes an approach to disease prevention and treatment that exploits the multiple distinct characteristics of each individual (in gene, environment, and lifestyle) to maximize effectiveness [1]. Early advances in precision medicine have largely occurred in oncology, where both diagnosis and treatment are increasingly based on genomic features. Better success rates from the treatment of her2-positive breast cancer [2] and EGFR-positive lung cancer [3] highlight the potential of precision medicine to lead to widespread changes in clinical practice. Growing interest is also reflected in new large-scale precision health projects, such as the NIH-sponsored Precision Medicine Initiative in the United States and the NHS-sponsored 100,000 Genomes project in Great Britain, as well as by citizen support for such ventures [4].

Enthusiasm for precision medicine can be seen across the spectrum of biomedical research, not only in oncology but also in cardiology [5], pulmonology [6], allergy [7], psychiatry [8], and public health [9]. Recently, a potential role for precision medicine in the ICU has also been discussed [10–18]. These discussions point to both the complexity and the ambiguity of critical illness syndromes as the fundamental justification for a precision approach. As inherently protean entities, common ICU conditions like sepsis, acute respiratory distress syndrome (ARDS), acute kidney injury, and delirium have manifestations and sequelae that are physiologically and anatomically diffuse, and that fluctuate over short periods of time. This leads to considerable heterogeneity among patients, and conditions in which a “one size fits all” approach to therapy can lead to widely divergent and even contradictory results [11, 19–21].

Important differences between patients—including differences in pathophysiology and variable risk of experiencing adverse events—are even seen in well-designed randomized clinical trials, in which patients are enrolled based on clinically defined syndromes [22]. This heterogeneity of treatment effect (HTE) may lead to significant variability in the overall benefit that patients stand to receive from the treatment under investigation. In these ways, current ICU therapy can be seen as *imprecise*, and thus has the potential to realize substantial gains from precision medicine approaches.

Recent studies suggest some ways in which complex ICU syndromes can be parsed into subtypes. Gene expression analysis has been used not only to differentiate sepsis from clinically similar non-infectious states [23], but also to distinguish molecularly defined subtypes of sepsis and septic shock [24–26], and to estimate how likely those subtypes are to respond to various treatments, such as corticosteroid therapy [27]. Targeted sequencing of candidate genes has been used to identify single nucleotide polymorphisms associated with either favorable or unfavorable outcomes in both sepsis [14, 28] and ARDS [29]. Genome-wide association studies (GWAS) of sepsis [30] and ARDS [31] have begun in earnest, and several pharmacogenes have also been identified that might add precision to the prescribing of drugs used in sepsis, such as norepinephrine, vasopressin, and corticosteroids [32].

Beyond genomics, greater precision has also emerged from other types of data. Clinical data have been used to identify four distinct subtypes of sepsis using machine learning techniques [33]. Serum biomarkers have shown promise in differentiating septic patients with ARDS from those without [34], and in classifying ARDS as due to either direct or indirect injury to the lungs [35]. Clinical data including vital sign measurements, ventilator settings, and laboratory data have been used alongside serum biomarker data to identify two physiologically distinct subtypes of ARDS [36]. These subtypes, also called endotypes, correlate not only with mortality, but also with a favorable response to specific therapeutic maneuvers such as ventilation with high PEEP [36] and conservative fluid management [37].

The recent identification of critical illness subtypes points to a growing need to harmonize them with precise therapies. But despite early gains in this new area of inquiry, the path to precision in the ICU is far from clear. In what follows we explore some of the challenges facing precision medicine in the ICU, along with specific measures to address these.

## Challenges of precision medicine

While there are compelling reasons to believe that critical care has much to gain from a precision medicine approach, such a pivot could be disruptive in many ways. ICU practice differs from that of the outpatient clinics where precision medicine programs are currently being developed; no template exists to guide its deployment in acute care settings. In defining a path to precision in the ICU, practitioners and researchers will confront the same challenges facing the precision medicine movement in general, along with obstacles unique to the fast-paced environment of the ICU.

Among the many possible challenges, we foresee three ways in which operationalizing precision medicine in the ICU may prove difficult. First, testing of individualized therapies implies ever-smaller cohorts of patients, meeting increasingly narrow eligibility criteria. Following the logic of precision medicine to its inevitable conclusion—namely that every patient is unique—leaves us with countless “*n* of 1” scenarios, a condition that, except in rare cases, is incompatible with current experimental approaches. This could lead to significantly longer recruitment times, increased complexity, and increased costs in carrying out clinical trials.

While this circumstance confronts all potential applications of precision medicine, the implications may be most evident in the ICU where multiple comorbidities, interactions between concurrent therapies, and rapidly changing physiologic states all enhance disease complexity. These added exigencies stand to complicate the time-sensitive task of recruiting acutely ill patients—many of whom lack decision-making capacity—into clinical trials.

Second, the process of developing and validating novel biomarkers to enhance treatment precision is long and onerous, with significant scientific, regulatory, and commercialization hurdles to be cleared. Despite more than 1000 publications on genetic polymorphisms in sepsis—a condition known to have important genetic determinants [38, 39]—none has led to the development of a so-called “companion diagnostic” test that would match patients with specific genotypes to a corresponding therapy [32]. Furthermore, to be useful in the ICU, diagnostic tests must be deployable at the point of care, with rapid turnaround times and low barriers to use. While today’s genome-wide technologies may be useful for biomarker discovery research, they are too slow for use in the ICU. Other biochemical, physiological, or clinical biomarkers may be more readily available, leading to greater utility in ICU settings.

Third, the vast quantities of ICU data needed to fuel precision medicine research are seldom readily available. Although most ICUs generate gigabytes of data each day, only a small fraction is accessible for research purposes [40]. Vital sign waveforms are often purged from bedside monitors at the time of ICU discharge. The use of electronic medical record (EMR) data is hindered by poor interoperability between platforms, legal and regulatory barriers to access, and questions of data validity and reliability [41]. Genome-wide data from the ICU remain relatively scarce, although genomic data generated for other purposes—such as clinically directed pharmacogenomic testing or personally directed sequencing done through direct-to-consumer products—have the potential to address this scarcity in part. Nonetheless, barriers to access and interpretability continue to limit the utility of these data. Critical care data infrastructure at the hospital and health system levels remains underdeveloped, undermining efforts to advance precision medicine in the ICU.

## Novel approaches to clinical trials

The precision medicine movement boldly confronts current practices in clinical research, in which large-scale randomized controlled trials (RCTs) recruit a heterogeneous group of patients in order to study the effect of an intervention. Within this framework, results are presented “on average” in a way that is antithetical to the precision ethos. With only a small minority of critical care RCTs yielding actionable evidence [19, 42], large-scale trials of heterogeneous patient populations are not achieving the goal of demonstrating the potential positive effect of the therapies studied. Changing funding priorities increasingly value innovative trial designs over expensive and time-consuming mega-trials. New trial designs are therefore needed [43–45].

One approach is to recruit more homogeneous groups of patients, with the hope that reducing population heterogeneity will increase the magnitude of the treatment effect [11, 19, 45, 46]. While the idea of studying an array of individual endotypes is daunting, a first approach is to start in earnest, by dividing a syndrome into just two distinct subtypes. This strategy was adopted by the MONARCS trial, which studied the effects of afelimomab—a monoclonal antibody to TNF-α—in the setting of septic shock [47]. The authors hypothesized that the study drug would be most effective in patients with high baseline levels of IL-6, and found that while the study population as a whole showed no difference in mortality, patients with elevated IL-6 levels realized a modest benefit.

As traditional RCTs become more precision oriented, we will be confronted with a more limited pool of patients, and will be forced to accept a smaller sample size, a longer time horizon for recruitment, or some degree of both. But by including only an enriched group of patients, a loss in statistical power due to smaller sample size may be offset by a gain in effect size.

Other potential solutions to the problem of narrow eligibility criteria involve entirely novel trial designs. One such design is the registry-based randomized controlled trial (RRCT), which capitalizes on data collected routinely for other reasons [48]. Patients being entered into an existing registry who meet prespecified enrollment criteria can be approached for consent and randomized; the screening, data capture, and outcomes measurement are all built in. The TASTE trial examining the use of thrombus aspiration in ST-segment elevation myocardial infarction used a RRCT design, resulting in substantial cost savings and rapid recruitment [49]. This strategy allows investigators to control costs, focus on patient recruitment, and still benefit from the power of randomization to draw the strongest possible conclusions about causation. Enhanced precision may then follow from enhanced recruitment that can more readily identify patients of a given subtype.

By screening vast groups of patients—theoretically those of an entire jurisdiction, health system, or investigator collaborative—RRCTs could systematically identify cohorts with specific characteristics, fulfilling the precision medicine mandate to study narrowly construed subgroups. Real-time data collection and analysis could augment this capability by enabling electronic surveillance systems (ESSs)—so-called “sniffers”—to rapidly identify suitable patients. The sniffer concept is perhaps best illustrated by the METRIC Data Mart, a clinical data warehouse built from the data of ICU patients at the Mayo Clinic [50]. METRIC receives EMR data uploads with a lag time of only 15 minutes, making it capable of rapid screening and detection of changing clinical states. METRIC sniffers have been used to expedite clinical research by identifying patients with acute kidney injury [51] and sepsis [52] in real time, so that study personnel can be notified quickly when patients meet enrollment criteria. In the case of the sepsis study, enrollment rates doubled after sniffer implementation.

A number of health jurisdictions are already positioned to turn existing registries into real-time alerting and reporting systems capable of supporting RRCT and ESS methodologies. In the Netherlands, nearly all of the 90 hospitals with ICUs contribute patient-level clinical data to a registry that now contains information on more than half a million ICU admissions [53]. The Australia and New Zealand Intensive Care Society (ANZICS) registry contains detailed clinical data on some 1.3 million admissions from more than 140 ICUs [54]. Nearly 20 countries have some form of ICU registry, with newer coalitions such as the International Forum for Acute Care Trialists (InFACT) raising the possibility of future international ICU registries [55].

Another new trial design for enrichment of treatment-responsive patient populations is the platform trial, so named because of the experimental platform used to conduct the study, which may persist thereafter and provide ongoing support for research and practice [56]. This design uses response-adaptive randomization to test a variety of treatments—either alone, in combination, or in different sequences—among a group of patients with a particular disease of interest. Heterogeneity within the study population is tackled explicitly, with precise subgroups defined at the outset. Patient randomization is weighted toward therapies that are likely to be more effective in subsequent clinical trials, based on Bayesian analysis of study data accrued to date. Treatments that prove less effective are eventually dropped, as are patient subgroups for which no effective treatment is identified. Only a handful of studies have as yet used a platform design, but the approach has the potential to rapidly and efficiently evaluate the harmonization of precision diagnostics with tailored therapies.

In critical care, a platform design is being used in a large European study of community-acquired pneumonia (AD-SCAP) to test various combinations of antibiotics, steroids, and ventilation strategies (ClinicalTrials.gov NCT02735707). The complex forecasting used in platform trials relies on vast quantities of data to probabilistically match patients with distinctive disease traits to the most promising therapies. These features suggest platform trials could serve as a first step toward precision medicine, in that they enroll a heterogeneous group of patients but allow for subtype-specific analysis in real time. This stands to lessen the chance that a clinical trial returns negative results due to differential treatment effects.

## Beyond genomics

The precision medicine movement is closely linked with genomics, and increasingly with other “omics” technologies, including epigenomics, proteomics, and metabolomics. These data have already been used in critical care research, including one study identifying metabolomic profiles that correlate with ICU survival [57], and others that investigate the role of the microbiome in critical illness [58]. However, most omics-based tests are still too slow to be useful in the ICU, and the data they generate do not readily inform clinical decision-making. Researchers must therefore look to other sources of data that better lend themselves to rapid collection and analysis.

Some investigators have looked at ways to downsize whole genome disease signatures to the smallest number of genes possible. Examples include an 11-gene signature to distinguish sepsis from non-infectious inflammatory states [23], and a two-gene signature to classify infection in children as either viral or bacterial in origin [59]. In one study, a gene expression signature was used to identify pediatric patients with sepsis who were at increased risk of acute kidney injury [60]. The serum protein levels of two of these gene products showed high sensitivity for identifying this condition. In another study [61], a 63-gene expression signature was identified that performed well in differentiating uncomplicated from complicated clinical trajectories following trauma. Based on this gene set, the investigators developed a novel assay using nanoString “molecular barcoding” technology that was able to generate an easily understandable composite score with a 12–24-hour turnaround time. Test performance surpassed that of traditional severity of illness scores such as the APACHE II score and the Injury Severity Score (ISS). These last examples show how diagnostic and prognostic signals identified using high-dimensional genomic data might be downsized to simpler tests with clinical applicability.

Beyond genomics, high-dimensional clinical data from the EMR may be useful in identifying groups of patients or even individuals with certain prognostic features. For example, detailed chronic comorbidity profiles could be used to more precisely prognosticate functional outcomes [62]. Such a system might capitalize on work being done to combine large volumes of clinical data with machine learning algorithms, in order to develop real-time predictive analytics capable of identifying meaningful subtypes. Novel physiologic markers might also prove useful, not only in making distinctions between endotypes but in providing continuous monitoring to characterize illness trajectory and track response to interventions. As one example, near-infrared spectroscopy (NIRS) technology may be useful in identifying specific subgroups of patients based on brain tissue oxygenation [63].

Routinely collected physiologic signals, such as continuous ECG tracings, may also prove useful in distinguishing patients who might otherwise appear similar. For example, the use of heart rate variability (HRV) metrics in critical care has been well studied [64] but is not currently used in clinical practice. With the potential to accurately prognosticate clinical deterioration and other intermediate outcomes, HRV monitoring could prove useful both in more precisely defining separate disease states and in detecting the dynamic transitions between these.

In one study, healthy participants receiving either enteral or parenteral nutrition experienced a significant decrease in HRV compared with those given an oral diet [65]. Those receiving parenteral nutrition also showed a significant change in monocyte gene expression after 72 hours of feeding. Another study examining HRV and genomic data together showed that trauma patients with either reduced HRV or specific polymorphisms in beta receptor genes had an increased risk of death following injury [66]. These studies suggest that surrogate markers of genomic features may be found within more readily accessible physiologic signals.

**Fig. 1:**
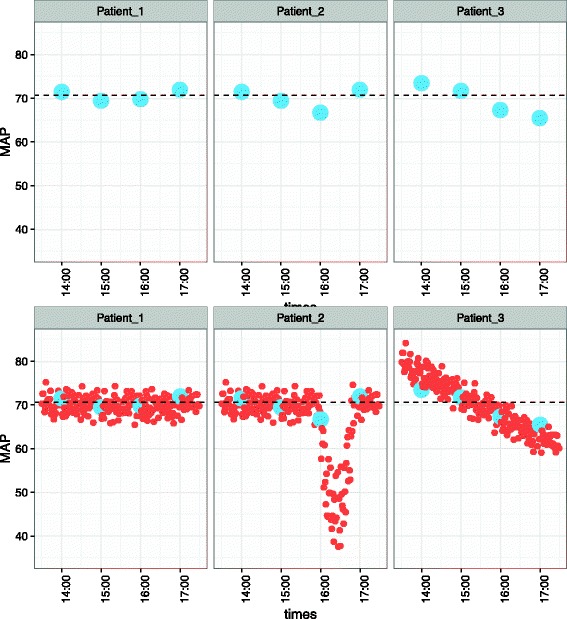
Effect of increasing quantities of data on revealing important distinctions. Four hours of mean arterial pressure (*MAP*) monitoring data (simulated) are shown for three different patients. With data recorded every hour (*top row*), MAP trajectories appear similar between patients, with a median MAP of 70 for all cases (*dashed line*). With data recorded every minute (*bottom row*), the median MAP is still 70 for all cases but important differences between patients become evident, with Patient 1 showing a relatively stable MAP, Patient 2 showing a precipitous drop in MAP at around 16:15, and Patient 3 showing a gradually decreasing MAP. In this case, the more granular data reveal differences between physiologic trajectories that were not evident from the sparse data or the median values

Ultimately, precision arises from our capacity to differentiate one patient’s manifestations of a syndrome from those of others. These distinctions arise from the increasing breadth and depth of data by which a patient’s condition can be characterized. Simply put, more data lead to better distinctions (Fig. 1). While genomic data will undoubtedly continue to play a role in precision critical care research, other more accessible sources of biomedical data will be vital in translating basic science insights into working tools for clinicians.

## Infrastructure and informatics for precision critical care

No matter what strategy is ultimately pursued, the path to precision in the ICU will run through large swaths of data. Large data sets—whether derived from new genomics platforms, physiologic waveforms, RCTs, or EMRs—are the cornerstone of precision medicine, underpinning new insights into the pathophysiology of critical illness, enabling the identification of distinct disease endotypes, and furnishing an infrastructure upon which to generate and test new hypotheses. With the costs of data storage falling, there is now an opportunity to harvest these data for use in research.

Some registries have already proven to be valuable commodities in critical care research. The MIMIC III database is one example of a large repository of ICU data that has been used to power retrospective analyses for hypothesis-generating research [67]. ICU data sets were also instrumental in the recent establishment and validation of the new definitions for sepsis [68]. However, collecting data is only a first step. Data must be validated, cleaned, stored, transformed, shared, protected, and analyzed, all of which requires dedicated informatics infrastructure and close coordination with informatics specialists. The collaborative task of developing bioinformatics platforms for precision medicine has begun in the field of cancer, with systems being designed to amalgamate genomic and clinical data, and to facilitate common analyses used in precision medicine research [69]. Similar systems for critical care research will have to account for ICU-specific data types (e.g., streaming physiologic waveforms), as well as the real-time exigencies of acute care practice.

The creation of data registries requires a significant investment, with dedicated computing resources needed to securely store data and ensure their protection. Database software tools are needed to structure and organize data, and to provide a portal of entry so that data can be contributed either manually or by automated transfer from other sources such as EMRs. Open source electronic data capture (EDC) tools may prove valuable in this regard, and have even been integrated with web-based randomization tools, demonstrating how informatics infrastructure could be used to support RRCTs [70]. Mobile interfaces for these applications could be useful in facilitating data collection at the bedside.

Maximizing the potential of large data sets will involve merging different data types in order to add clinical context to biological data. It will also require that data from different sites are coanalyzed to add statistical power to “small *n*” studies of disease subtypes. The sharing of biomedical data has become challenging as the sheer volume of data skyrockets, and as concern for security increases. Explicit data management strategies must be developed to protect sensitive health information, while respecting the tenets of data sharing, open science, and collaboration between research groups. Data stewardship plans must address issues of access, security, and accountability, and must have protocols in place for anonymizing patient data, creating research ethics protocols, and generating data sharing agreements. Compliance with regulatory requirements should be considered so that data from investigator-initiated and industry-sponsored trials can be intermingled, and so that hospitals can confidently create linkages for data uploads. Ultimately the path of least resistance may be a flipped model of data analytics in which the analysis is brought to the data, obviating the need to push data around. This model would address potential concerns around relinquishing control over data, ensuring data protection, and mitigating risks from technical failures and unscheduled downtime.

Regardless of whether data are migrated to a central location, or analytic tools are brought to local data stores, the task of processing data from diverse sources will benefit from the development of a formal ontology of critical care concepts. An ontology is a controlled vocabulary specifying a set of terms and the relationships between them, providing an essential ground truth to mediate the merging of data elements from different sources [71]. By mapping terms to a common ontology, data from disparate sites—where EMRs, bedside monitors, and genomic platforms might differ—can be coanalyzed. Numerous biomedical ontologies have been generated, including some dealing with precision medicine concepts [71, 72]. Work in this area should include clinicians, researchers, ethicists, and patient representatives, so that informatics tools meet the closely linked needs of providing EMR-enabled patient care and conducting patient-centered research.

## Costs and opportunities

Although the cost of sequencing and other genomics technologies continues to fall, research in precision medicine will undoubtedly be expensive, with more studies needed to answer more precise questions. The approaches described, such as those in which RRCTs are used to study a small number of endotypes, may represent a useful starting point. However, important questions remain around how best to implement precision-based testing and treatment. To what extent should existing strategies that confer a marginal benefit to a large group of people be supplanted by those that offer better overall results but to a select few [73]?

One illustration of some of the tensions around large-scale implementation of precision medicine is the decision by the US Center for Medicare and Medicaid Services (CMS) not to reimburse pharmacogenomic-guided prescribing of warfarin. Although patients with rare genomic variants likely benefit from this approach, clinical outcomes remain equivalent to conventional prescribing when the testing is deployed across large groups [73, 74]. Conventional warfarin prescribing may therefore be cost-effective overall, but needlessly detrimental to a minority of patients in whom preventable bleeding or thrombotic complications may ensue.

These circumstances have parallels with critical care practice, in which considerable resources are expended to provide patients with treatment that may be useful in some cases, but ineffective or harmful in others. Precision methods may prove useful in the early identification of patients for whom a particular therapy will be ineffective (so-called “nonresponders”), which may lead to the avoidance of harms, fewer delays to treatment, and a more effective allocation of resources.

Closely related to the concepts of ineffective or harmful treatments is the notion that some treatments in the ICU are in fact unwanted altogether. Precision critical care may thus involve not only matching treatments with the patients most likely to benefit, but also ensuring that treatments are aligned with patient preferences. This can be done immediately—and at negligible cost—by making sure that aggressive care is provided only to those who want it, especially at the end of life [75]. While some aspects of precision medicine research and practice may prove to be costly, so too is the sustained use of invasive life support among patients who will not ultimately benefit or who might wish to forgo such treatment altogether.

## Conclusion

A deluge of data accompanies the tremendous complexity and heterogeneity of critical illness. These conditions make critical care fertile ground for an exploration of precision medicine approaches to research and practice. Change may be incremental rather than wholesale, in which small proof-of-concept studies demonstrate the viability of precision critical care. Novel trial designs will be needed to more efficiently enroll patients with narrowly defined syndrome subtypes. Both genomic and nongenomic data must be coopted to derive new insights into critical care endotypes and rapidly identify patients at the bedside. These tasks must be supported by a robust data infrastructure developed by clinicians, researchers, and data scientists.

Precision medicine defines an approach in which the clinician comes to the bedside seeking to interrogate and understand their patient’s unique physiology. While the terminology may be new, this sensibility harkens back to the earliest incarnations of critical care, and remains as its core today.

## Abbreviations

ANZICS: Australia and New Zealand Intensive Care Society
ARDS: Acute respiratory distress syndrome
CMS: Center for Medicare and Medicaid Services
ECG: Electrocardiography
EDC: Electronic data capture
EMR: Electronic medical record
ESS: Electronic surveillance system
GWAS: Genome-wide association study
HRV: Heart rate variability
HTE: Heterogeneity of treatment effect
ICU: Intensive care unit
InFACT: International Forum for Acute Care Trialists
ISS: Injury Severity Score
NHS: National Health Service
NIH: National Institutes of Health
NIRS: Near-infrared spectroscopy
PEEP: Positive end-expiratory pressure
RCT: Randomized controlled trial
RRCT: Registry randomized controlled trial
TNF: Tumor necrosis factor
